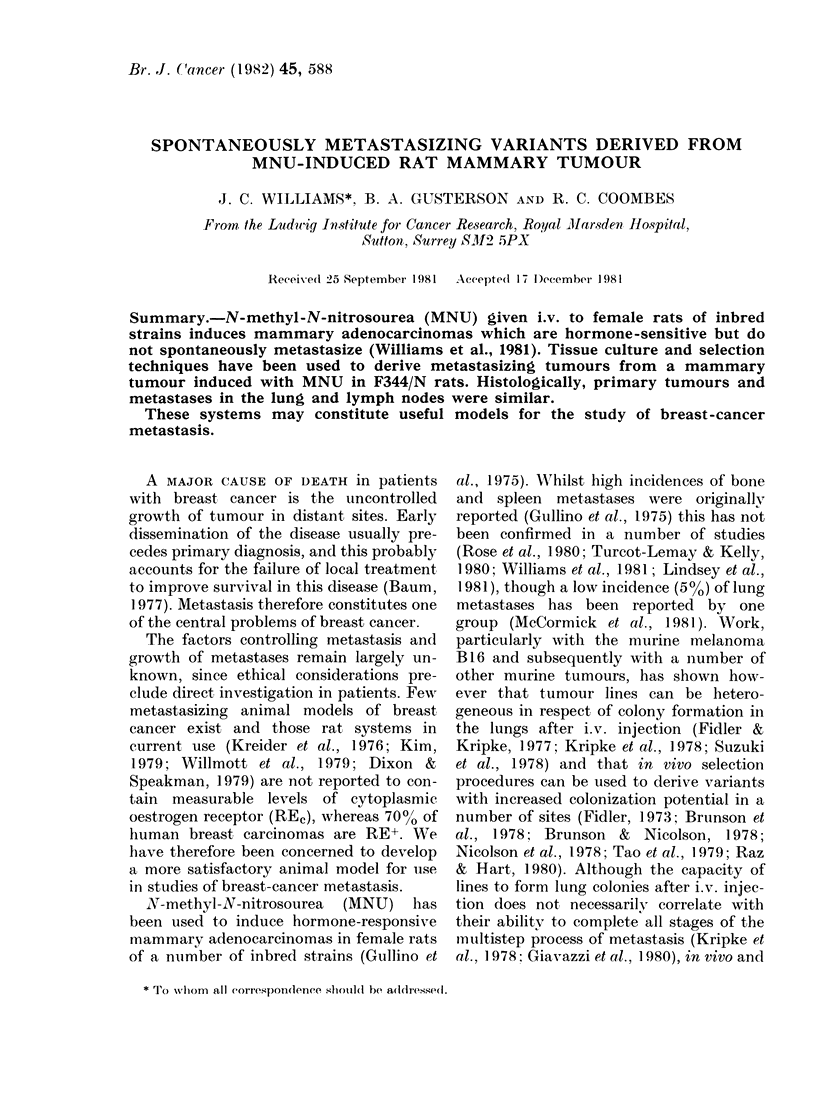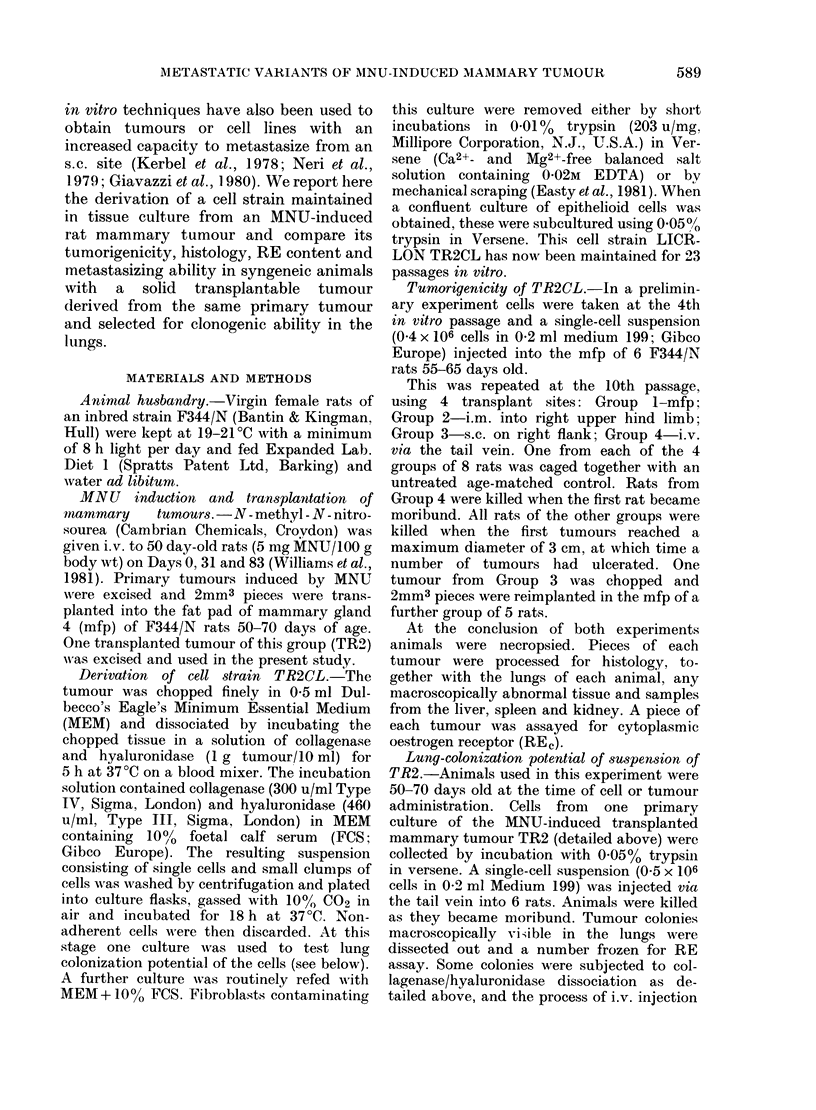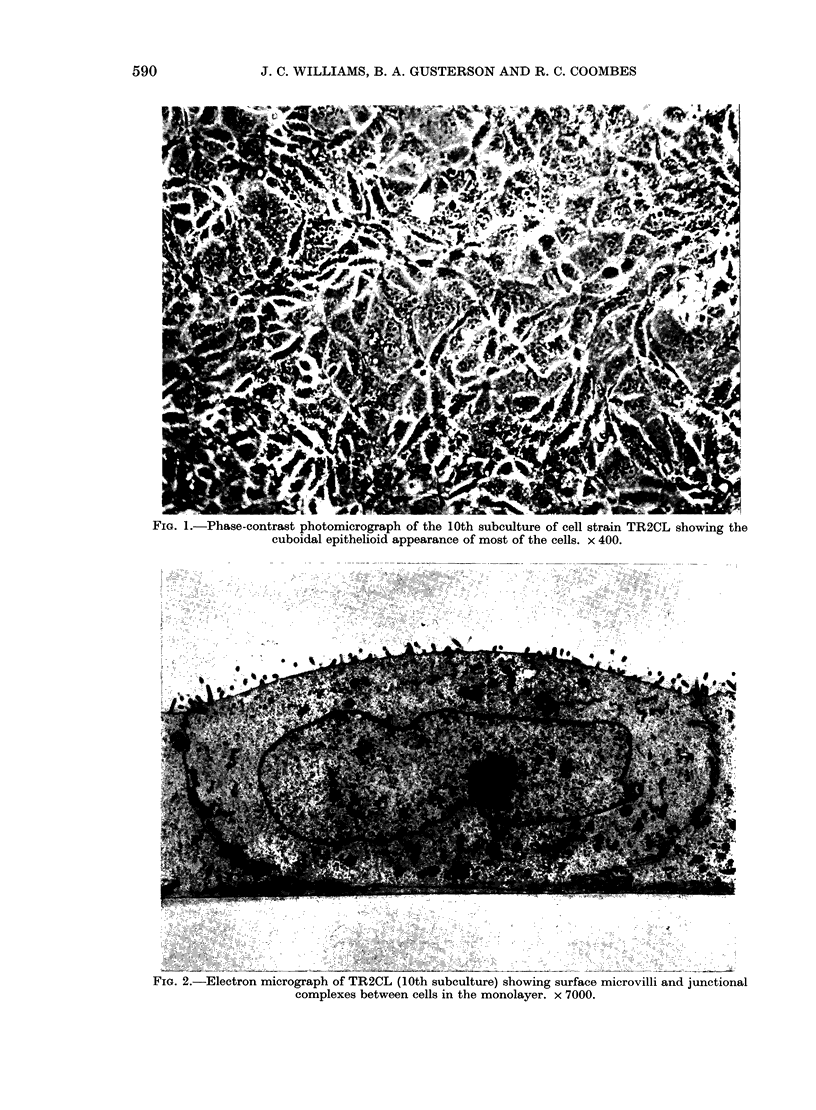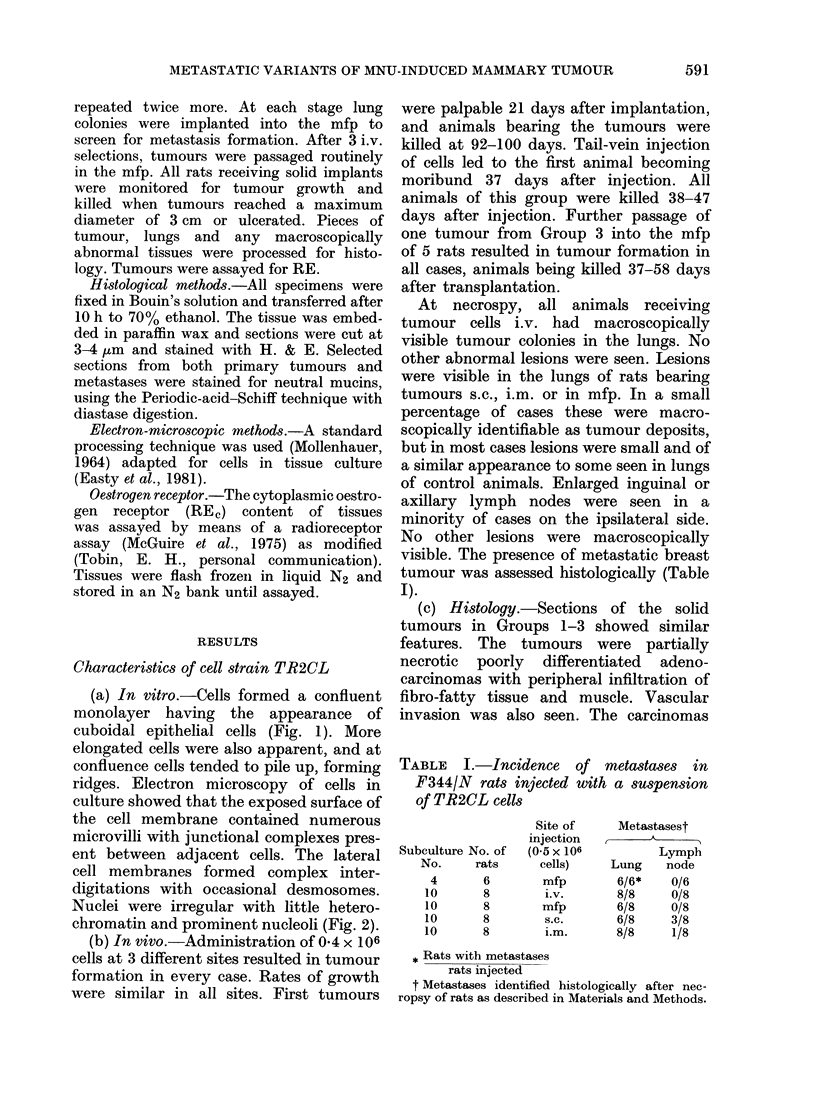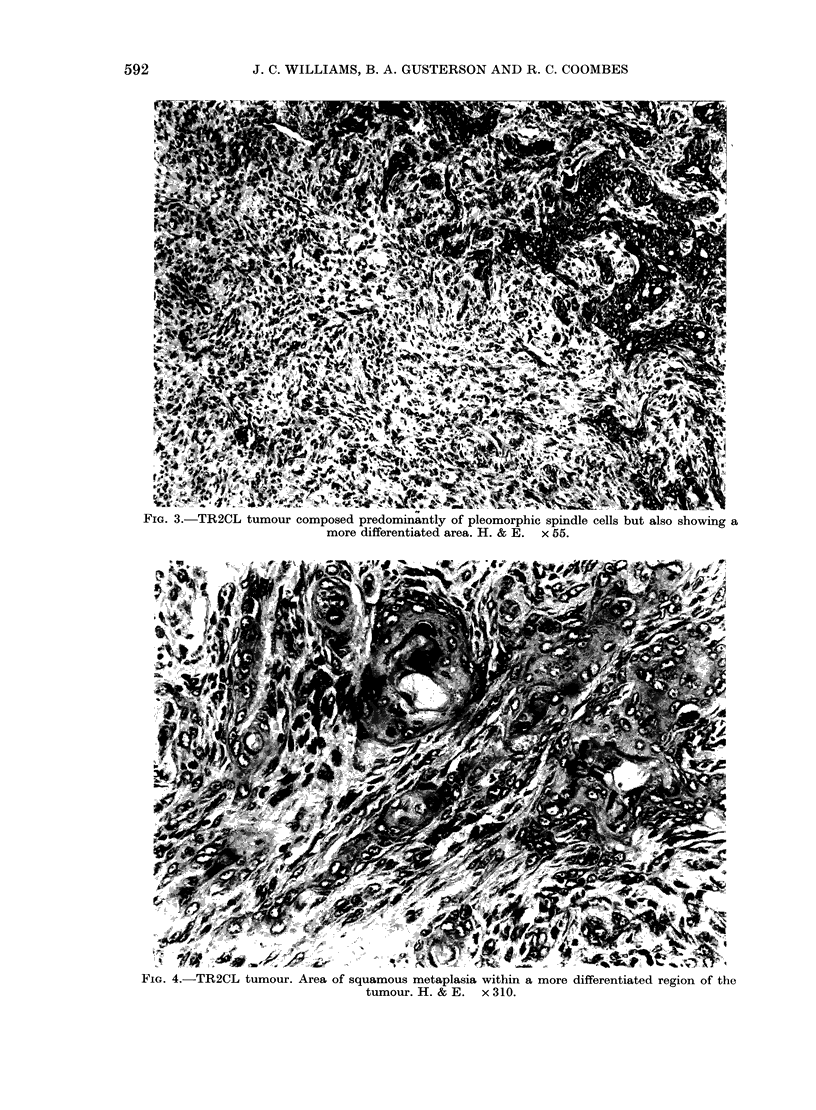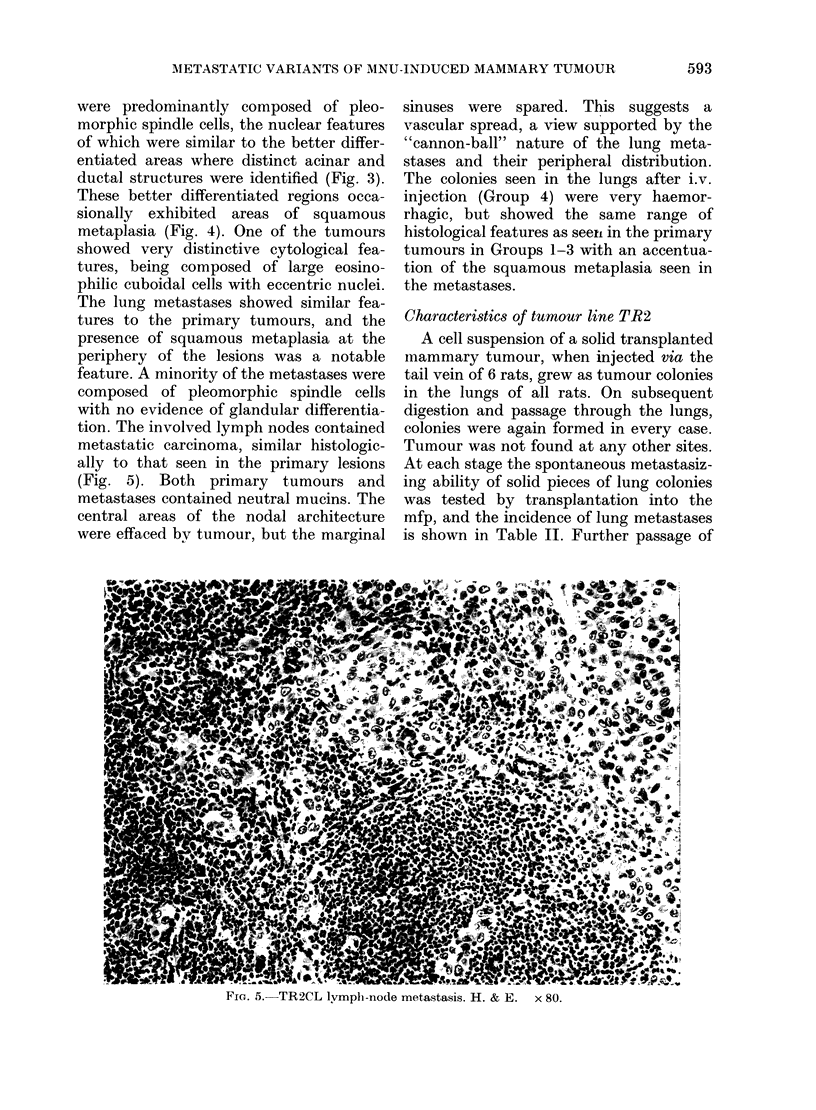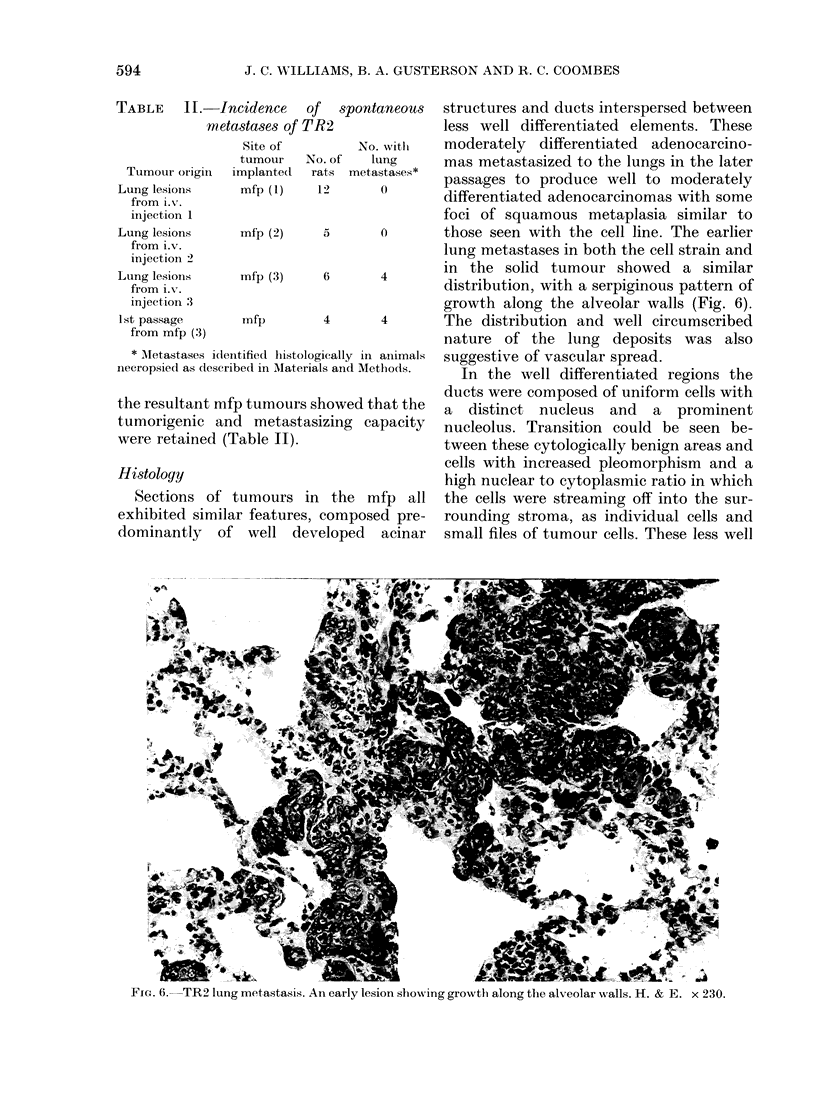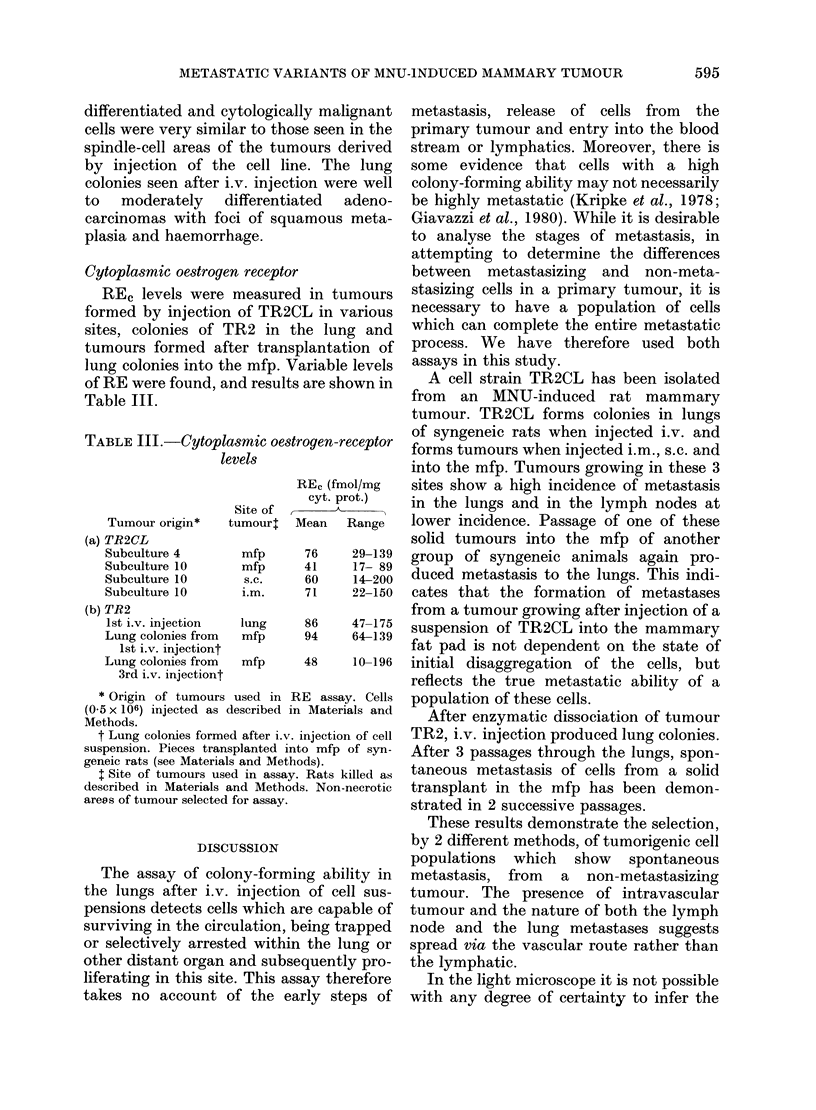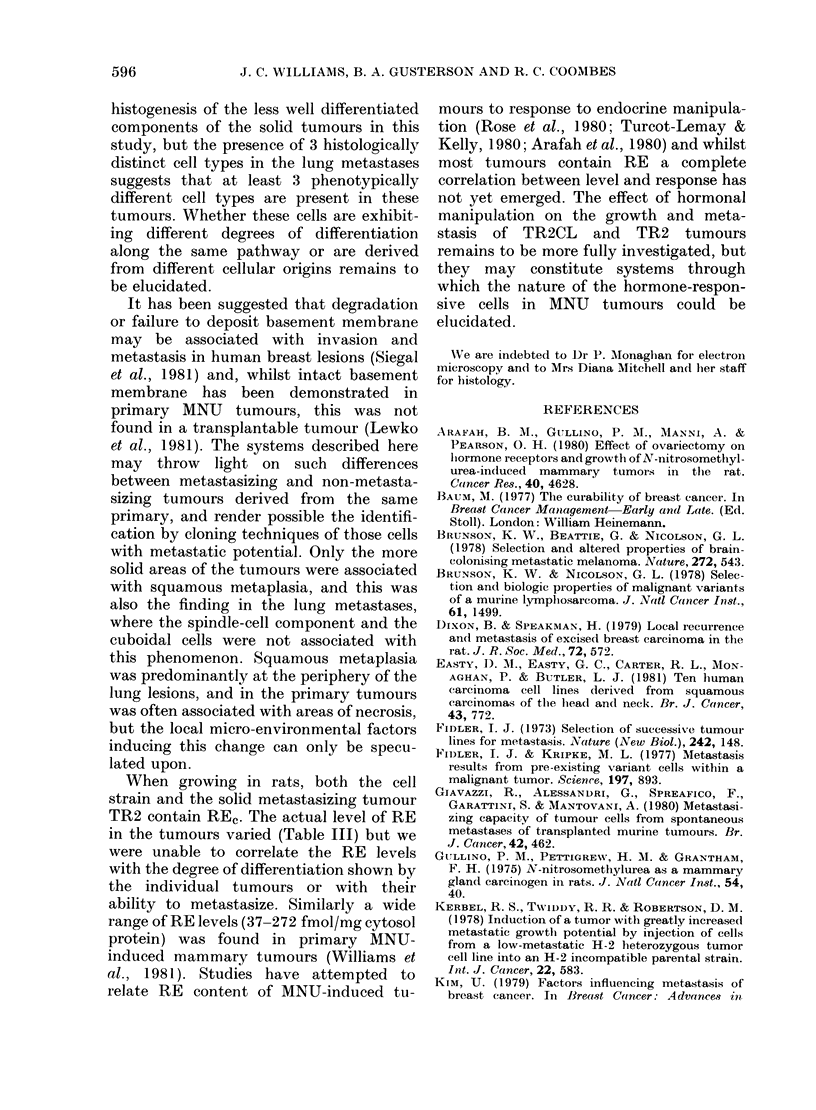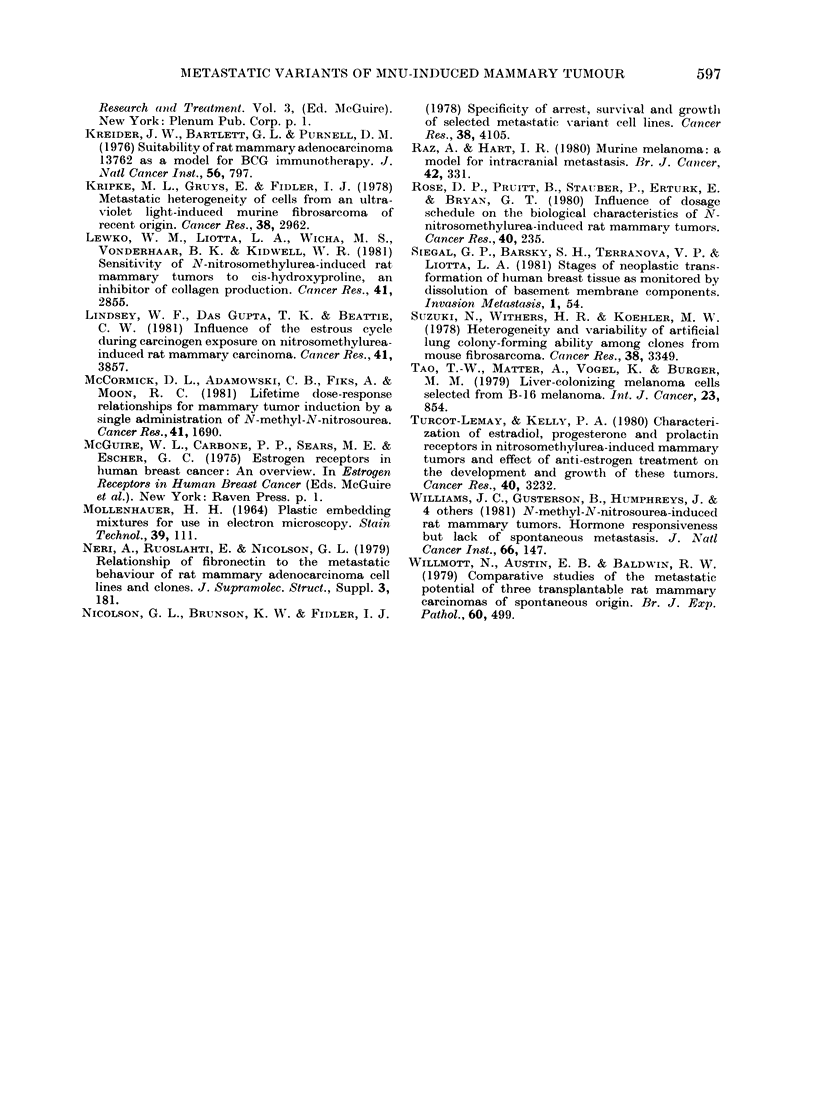# Spontaneously metastasizing variants derived from MNU-induced rat mammary tumour.

**DOI:** 10.1038/bjc.1982.96

**Published:** 1982-04

**Authors:** J. C. Williams, B. A. Gusterson, R. C. Coombes

## Abstract

**Images:**


Br. J. Cancer (1982) 45, 588

### SPONTANEOUSLY METASTASIZING VARIANTS DERIVED FROM MNU-INDUCED RAT MAMMARY TUMOUR

J. C. WILLIAMS*, B. A. GUSTERSON AND R. C. COOMBES

*From the Ludwig Institute for Cancer Research, Royal Marsden Hospital, Sutton, Surrey SM2 5PX*

Received 25 September 1981 Accepted 17 December 1981

**Summary.—*N*-methyl-*N*-nitrosourea (MNU) given i.v. to female rats of inbred strains induces mammary adenocarcinomas which are hormone-sensitive but do not spontaneously metastasize (Williams et al., 1981). Tissue culture and selection techniques have been used to derive metastasizing tumours from a mammary tumour induced with MNU in F344/N rats. Histologically, primary tumours and metastases in the lung and lymph nodes were similar.**

**These systems may constitute useful models for the study of breast-cancer metastasis.**

A MAJOR CAUSE OF DEATH in patients with breast cancer is the uncontrolled growth of tumour in distant sites. Early dissemination of the disease usually precedes primary diagnosis, and this probably accounts for the failure of local treatment to improve survival in this disease (Baum, 1977). Metastasis therefore constitutes one of the central problems of breast cancer.

The factors controlling metastasis and growth of metastases remain largely unknown, since ethical considerations preclude direct investigation in patients. Few metastasizing animal models of breast cancer exist and those rat systems in current use (Kreider *et al.*, 1976; Kim, 1979; Willmott *et al.*, 1979; Dixon & Speakman, 1979) are not reported to contain measurable levels of cytoplasmic oestrogen receptor ($RE_c$), whereas 70% of human breast carcinomas are $RE^+$. We have therefore been concerned to develop a more satisfactory animal model for use in studies of breast-cancer metastasis.

*N*-methyl-*N*-nitrosourea (MNU) has been used to induce hormone-responsive mammary adenocarcinomas in female rats of a number of inbred strains (Gullino *et al.*, 1975). Whilst high incidences of bone and spleen metastases were originally reported (Gullino *et al.*, 1975) this has not been confirmed in a number of studies (Rose *et al.*, 1980; Turcot-Lemay & Kelly, 1980; Williams *et al.*, 1981; Lindsey *et al.*, 1981), though a low incidence (5%) of lung metastases has been reported by one group (McCormick *et al.*, 1981). Work, particularly with the murine melanoma B16 and subsequently with a number of other murine tumours, has shown however that tumour lines can be heterogeneous in respect of colony formation in the lungs after i.v. injection (Fidler & Kripke, 1977; Kripke *et al.*, 1978; Suzuki *et al.*, 1978) and that *in vivo* selection procedures can be used to derive variants with increased colonization potential in a number of sites (Fidler, 1973; Brunson *et al.*, 1978; Brunson & Nicolson, 1978; Nicolson *et al.*, 1978; Tao *et al.*, 1979; Raz & Hart, 1980). Although the capacity of lines to form lung colonies after i.v. injection does not necessarily correlate with their ability to complete all stages of the multistep process of metastasis (Kripke *et al.*, 1978; Giavazzi *et al.*, 1980), *in vivo* and

* To whom all correspondence should be addressed.

*in vitro* techniques have also been used to obtain tumours or cell lines with an increased capacity to metastasize from an s.c. site (Kerbel *et al.*, 1978; Neri *et al.*, 1979; Giavazzi *et al.*, 1980). We report here the derivation of a cell strain maintained in tissue culture from an MNU-induced rat mammary tumour and compare its tumorigenicity, histology, RE content and metastasizing ability in syngeneic animals with a solid transplantable tumour derived from the same primary tumour and selected for clonogenic ability in the lungs.

### MATERIALS AND METHODS

*Animal husbandry.*—Virgin female rats of an inbred strain F344/N (Bantin & Kingman, Hull) were kept at 19–21 °C with a minimum of 8 h light per day and fed Expanded Lab. Diet 1 (Spratts Patent Ltd, Barking) and water *ad libitum*.

*MNU induction and transplantation of mammary tumours.*—*N*-methyl-*N*-nitrosourea (Cambrian Chemicals, Croydon) was given i.v. to 50 day-old rats (5 mg MNU/100 g body wt) on Days 0, 31 and 83 (Williams *et al.*, 1981). Primary tumours induced by MNU were excised and 2mm$^3$ pieces were transplanted into the fat pad of mammary gland 4 (mfp) of F344/N rats 50–70 days of age. One transplanted tumour of this group (TR2) was excised and used in the present study.

*Derivation of cell strain TR2CL.*—The tumour was chopped finely in 0·5 ml Dulbecco's Eagle's Minimum Essential Medium (MEM) and dissociated by incubating the chopped tissue in a solution of collagenase and hyaluronidase (1 g tumour/10 ml) for 5 h at 37 °C on a blood mixer. The incubation solution contained collagenase (300 u/ml Type IV, Sigma, London) and hyaluronidase (460 u/ml, Type III, Sigma, London) in MEM containing 10% foetal calf serum (FCS; Gibco Europe). The resulting suspension consisting of single cells and small clumps of cells was washed by centrifugation and plated into culture flasks, gassed with 10% $CO_2$ in air and incubated for 18 h at 37 °C. Non-adherent cells were then discarded. At this stage one culture was used to test lung colonization potential of the cells (see below). A further culture was routinely refed with MEM + 10% FCS. Fibroblasts contaminating this culture were removed either by short incubations in 0·01% trypsin (203 u/mg, Millipore Corporation, N.J., U.S.A.) in Versene ($Ca^{2+}$- and $Mg^{2+}$-free balanced salt solution containing 0·02M EDTA) or by mechanical scraping (Easty *et al.*, 1981). When a confluent culture of epithelioid cells was obtained, these were subcultured using 0·05% trypsin in Versene. This cell strain LICR-LON TR2CL has now been maintained for 23 passages *in vitro*.

*Tumorigenicity of TR2CL.*—In a preliminary experiment cells were taken at the 4th *in vitro* passage and a single-cell suspension ($0{\cdot}4 \times 10^6$ cells in 0·2 ml medium 199; Gibco Europe) injected into the mfp of 6 F344/N rats 55–65 days old.

This was repeated at the 10th passage, using 4 transplant sites: Group 1–mfp; Group 2—i.m. into right upper hind limb; Group 3—s.c. on right flank; Group 4—i.v. *via* the tail vein. One from each of the 4 groups of 8 rats was caged together with an untreated age-matched control. Rats from Group 4 were killed when the first rat became moribund. All rats of the other groups were killed when the first tumours reached a maximum diameter of 3 cm, at which time a number of tumours had ulcerated. One tumour from Group 3 was chopped and 2mm$^3$ pieces were reimplanted in the mfp of a further group of 5 rats.

At the conclusion of both experiments animals were necropsied. Pieces of each tumour were processed for histology, together with the lungs of each animal, any macroscopically abnormal tissue and samples from the liver, spleen and kidney. A piece of each tumour was assayed for cytoplasmic oestrogen receptor ($RE_c$).

*Lung-colonization potential of suspension of TR2.*—Animals used in this experiment were 50–70 days old at the time of cell or tumour administration. Cells from one primary culture of the MNU-induced transplanted mammary tumour TR2 (detailed above) were collected by incubation with 0·05% trypsin in versene. A single-cell suspension ($0{\cdot}5 \times 10^6$ cells in 0·2 ml Medium 199) was injected *via* the tail vein into 6 rats. Animals were killed as they became moribund. Tumour colonies macroscopically visible in the lungs were dissected out and a number frozen for RE assay. Some colonies were subjected to collagenase/hyaluronidase dissociation as detailed above, and the process of i.v. injection

FIG. 1.—Phase-contrast photomicrograph of the 10th subculture of cell strain TR2CL showing the cuboidal epithelioid appearance of most of the cells. ×400.

FIG. 2.—Electron micrograph of TR2CL (10th subculture) showing surface microvilli and junctional complexes between cells in the monolayer. ×7000.

repeated twice more. At each stage lung colonies were implanted into the mfp to screen for metastasis formation. After 3 i.v. selections, tumours were passaged routinely in the mfp. All rats receiving solid implants were monitored for tumour growth and killed when tumours reached a maximum diameter of 3 cm or ulcerated. Pieces of tumour, lungs and any macroscopically abnormal tissues were processed for histology. Tumours were assayed for RE.

*Histological methods.*—All specimens were fixed in Bouin's solution and transferred after 10 h to 70% ethanol. The tissue was embedded in paraffin wax and sections were cut at 3–4 $\mu$m and stained with H. & E. Selected sections from both primary tumours and metastases were stained for neutral mucins, using the Periodic-acid–Schiff technique with diastase digestion.

*Electron-microscopic methods.*—A standard processing technique was used (Mollenhauer, 1964) adapted for cells in tissue culture (Easty *et al.*, 1981).

*Oestrogen receptor.*—The cytoplasmic oestrogen receptor ($RE_c$) content of tissues was assayed by means of a radioreceptor assay (McGuire *et al.*, 1975) as modified (Tobin, E. H., personal communication). Tissues were flash frozen in liquid $N_2$ and stored in an $N_2$ bank until assayed.

### RESULTS

### *Characteristics of cell strain TR2CL*

(a) *In vitro.*—Cells formed a confluent monolayer having the appearance of cuboidal epithelial cells (Fig. 1). More elongated cells were also apparent, and at confluence cells tended to pile up, forming ridges. Electron microscopy of cells in culture showed that the exposed surface of the cell membrane contained numerous microvilli with junctional complexes present between adjacent cells. The lateral cell membranes formed complex interdigitations with occasional desmosomes. Nuclei were irregular with little heterochromatin and prominent nucleoli (Fig. 2).

(b) *In vivo.*—Administration of $0{\cdot}4 \times 10^6$ cells at 3 different sites resulted in tumour formation in every case. Rates of growth were similar in all sites. First tumours were palpable 21 days after implantation, and animals bearing the tumours were killed at 92–100 days. Tail-vein injection of cells led to the first animal becoming moribund 37 days after injection. All animals of this group were killed 38–47 days after injection. Further passage of one tumour from Group 3 into the mfp of 5 rats resulted in tumour formation in all cases, animals being killed 37–58 days after transplantation.

At necrospy, all animals receiving tumour cells i.v. had macroscopically visible tumour colonies in the lungs. No other abnormal lesions were seen. Lesions were visible in the lungs of rats bearing tumours s.c., i.m. or in mfp. In a small percentage of cases these were macroscopically identifiable as tumour deposits, but in most cases lesions were small and of a similar appearance to some seen in lungs of control animals. Enlarged inguinal or axillary lymph nodes were seen in a minority of cases on the ipsilateral side. No other lesions were macroscopically visible. The presence of metastatic breast tumour was assessed histologically (Table I).

(c) *Histology.*—Sections of the solid tumours in Groups 1–3 showed similar features. The tumours were partially necrotic poorly differentiated adenocarcinomas with peripheral infiltration of fibro-fatty tissue and muscle. Vascular invasion was also seen. The carcinomas

TABLE I.—*Incidence of metastases in F344/N rats injected with a suspension of TR2CL cells*

| Subculture No. | No. of rats | Site of injection ($0{\cdot}5 \times 10^6$ cells) | Metastases† Lung | Metastases† Lymph node |
|---|---|---|---|---|
| 4 | 6 | mfp | 6/6* | 0/6 |
| 10 | 8 | i.v. | 8/8 | 0/8 |
| 10 | 8 | mfp | 6/8 | 0/8 |
| 10 | 8 | s.c. | 6/8 | 3/8 |
| 10 | 8 | i.m. | 8/8 | 1/8 |

* Rats with metastases / rats injected

† Metastases identified histologically after necropsy of rats as described in Materials and Methods.

FIG. 3.—TR2CL tumour composed predominantly of pleomorphic spindle cells but also showing a more differentiated area. H. & E. ×55.

FIG. 4.—TR2CL tumour. Area of squamous metaplasia within a more differentiated region of the tumour. H. & E. ×310.

were predominantly composed of pleomorphic spindle cells, the nuclear features of which were similar to the better differentiated areas where distinct acinar and ductal structures were identified (Fig. 3). These better differentiated regions occasionally exhibited areas of squamous metaplasia (Fig. 4). One of the tumours showed very distinctive cytological features, being composed of large eosinophilic cuboidal cells with eccentric nuclei. The lung metastases showed similar features to the primary tumours, and the presence of squamous metaplasia at the periphery of the lesions was a notable feature. A minority of the metastases were composed of pleomorphic spindle cells with no evidence of glandular differentiation. The involved lymph nodes contained metastatic carcinoma, similar histologically to that seen in the primary lesions (Fig. 5). Both primary tumours and metastases contained neutral mucins. The central areas of the nodal architecture were effaced by tumour, but the marginal sinuses were spared. This suggests a vascular spread, a view supported by the "cannon-ball" nature of the lung metastases and their peripheral distribution. The colonies seen in the lungs after i.v. injection (Group 4) were very haemorrhagic, but showed the same range of histological features as seen in the primary tumours in Groups 1–3 with an accentuation of the squamous metaplasia seen in the metastases.

*Characteristics of tumour line TR2*

A cell suspension of a solid transplanted mammary tumour, when injected *via* the tail vein of 6 rats, grew as tumour colonies in the lungs of all rats. On subsequent digestion and passage through the lungs, colonies were again formed in every case. Tumour was not found at any other sites. At each stage the spontaneous metastasizing ability of solid pieces of lung colonies was tested by transplantation into the mfp, and the incidence of lung metastases is shown in Table II. Further passage of

FIG. 5.—TR2CL lymph-node metastasis. H. & E. ×80.

TABLE II.—*Incidence of spontaneous metastases of TR2*

| Tumour origin | Site of tumour implanted | No. of rats | No. with lung metastases* |
|---|---|---|---|
| Lung lesions from i.v. injection 1 | mfp (1) | 12 | 0 |
| Lung lesions from i.v. injection 2 | mfp (2) | 5 | 0 |
| Lung lesions from i.v. injection 3 | mfp (3) | 6 | 4 |
| 1st passage from mfp (3) | mfp | 4 | 4 |

* Metastases identified histologically in animals necropsied as described in Materials and Methods.

the resultant mfp tumours showed that the tumorigenic and metastasizing capacity were retained (Table II).

*Histology*

Sections of tumours in the mfp all exhibited similar features, composed predominantly of well developed acinar structures and ducts interspersed between less well differentiated elements. These moderately differentiated adenocarcinomas metastasized to the lungs in the later passages to produce well to moderately differentiated adenocarcinomas with some foci of squamous metaplasia similar to those seen with the cell line. The earlier lung metastases in both the cell strain and in the solid tumour showed a similar distribution, with a serpiginous pattern of growth along the alveolar walls (Fig. 6). The distribution and well circumscribed nature of the lung deposits was also suggestive of vascular spread.

In the well differentiated regions the ducts were composed of uniform cells with a distinct nucleus and a prominent nucleolus. Transition could be seen between these cytologically benign areas and cells with increased pleomorphism and a high nuclear to cytoplasmic ratio in which the cells were streaming off into the surrounding stroma, as individual cells and small files of tumour cells. These less well

FIG. 6.—TR2 lung metastasis. An early lesion showing growth along the alveolar walls. H. & E. ×230.

differentiated and cytologically malignant cells were very similar to those seen in the spindle-cell areas of the tumours derived by injection of the cell line. The lung colonies seen after i.v. injection were well to moderately differentiated adenocarcinomas with foci of squamous metaplasia and haemorrhage.

*Cytoplasmic oestrogen receptor*

$RE_C$ levels were measured in tumours formed by injection of TR2CL in various sites, colonies of TR2 in the lung and tumours formed after transplantation of lung colonies into the mfp. Variable levels of RE were found, and results are shown in Table III.

TABLE III.—*Cytoplasmic oestrogen-receptor levels*

| Tumour origin* | Site of tumour‡ | $RE_c$ (fmol/mg cyt. prot.) Mean | Range |
|---|---|---|---|
| (a) *TR2CL* | | | |
| Subculture 4 | mfp | 76 | 29–139 |
| Subculture 10 | mfp | 41 | 17– 89 |
| Subculture 10 | s.c. | 60 | 14–200 |
| Subculture 10 | i.m. | 71 | 22–150 |
| (b) *TR2* | | | |
| 1st i.v. injection | lung | 86 | 47–175 |
| Lung colonies from 1st i.v. injection† | mfp | 94 | 64–139 |
| Lung colonies from 3rd i.v. injection† | mfp | 48 | 10–196 |

* Origin of tumours used in RE assay. Cells ($0\cdot5 \times 10^6$) injected as described in Materials and Methods.

† Lung colonies formed after i.v. injection of cell suspension. Pieces transplanted into mfp of syngeneic rats (see Materials and Methods).

‡ Site of tumours used in assay. Rats killed as described in Materials and Methods. Non-necrotic areas of tumour selected for assay.

### DISCUSSION

The assay of colony-forming ability in the lungs after i.v. injection of cell suspensions detects cells which are capable of surviving in the circulation, being trapped or selectively arrested within the lung or other distant organ and subsequently proliferating in this site. This assay therefore takes no account of the early steps of metastasis, release of cells from the primary tumour and entry into the blood stream or lymphatics. Moreover, there is some evidence that cells with a high colony-forming ability may not necessarily be highly metastatic (Kripke *et al.*, 1978; Giavazzi *et al.*, 1980). While it is desirable to analyse the stages of metastasis, in attempting to determine the differences between metastasizing and non-metastasizing cells in a primary tumour, it is necessary to have a population of cells which can complete the entire metastatic process. We have therefore used both assays in this study.

A cell strain TR2CL has been isolated from an MNU-induced rat mammary tumour. TR2CL forms colonies in lungs of syngeneic rats when injected i.v. and forms tumours when injected i.m., s.c. and into the mfp. Tumours growing in these 3 sites show a high incidence of metastasis in the lungs and in the lymph nodes at lower incidence. Passage of one of these solid tumours into the mfp of another group of syngeneic animals again produced metastasis to the lungs. This indicates that the formation of metastases from a tumour growing after injection of a suspension of TR2CL into the mammary fat pad is not dependent on the state of initial disaggregation of the cells, but reflects the true metastatic ability of a population of these cells.

After enzymatic dissociation of tumour TR2, i.v. injection produced lung colonies. After 3 passages through the lungs, spontaneous metastasis of cells from a solid transplant in the mfp has been demonstrated in 2 successive passages.

These results demonstrate the selection, by 2 different methods, of tumorigenic cell populations which show spontaneous metastasis, from a non-metastasizing tumour. The presence of intravascular tumour and the nature of both the lymph node and the lung metastases suggests spread *via* the vascular route rather than the lymphatic.

In the light microscope it is not possible with any degree of certainty to infer the histogenesis of the less well differentiated components of the solid tumours in this study, but the presence of 3 histologically distinct cell types in the lung metastases suggests that at least 3 phenotypically different cell types are present in these tumours. Whether these cells are exhibiting different degrees of differentiation along the same pathway or are derived from different cellular origins remains to be elucidated.

It has been suggested that degradation or failure to deposit basement membrane may be associated with invasion and metastasis in human breast lesions (Siegal *et al.*, 1981) and, whilst intact basement membrane has been demonstrated in primary MNU tumours, this was not found in a transplantable tumour (Lewko *et al.*, 1981). The systems described here may throw light on such differences between metastasizing and non-metastasizing tumours derived from the same primary, and render possible the identification by cloning techniques of those cells with metastatic potential. Only the more solid areas of the tumours were associated with squamous metaplasia, and this was also the finding in the lung metastases, where the spindle-cell component and the cuboidal cells were not associated with this phenomenon. Squamous metaplasia was predominantly at the periphery of the lung lesions, and in the primary tumours was often associated with areas of necrosis, but the local micro-environmental factors inducing this change can only be speculated upon.

When growing in rats, both the cell strain and the solid metastasizing tumour TR2 contain $RE_C$. The actual level of RE in the tumours varied (Table III) but we were unable to correlate the RE levels with the degree of differentiation shown by the individual tumours or with their ability to metastasize. Similarly a wide range of RE levels (37–272 fmol/mg cytosol protein) was found in primary MNU-induced mammary tumours (Williams *et al.*, 1981). Studies have attempted to relate RE content of MNU-induced tumours to response to endocrine manipulation (Rose *et al.*, 1980; Turcot-Lemay & Kelly, 1980; Arafah *et al.*, 1980) and whilst most tumours contain RE a complete correlation between level and response has not yet emerged. The effect of hormonal manipulation on the growth and metastasis of TR2CL and TR2 tumours remains to be more fully investigated, but they may constitute systems through which the nature of the hormone-responsive cells in MNU tumours could be elucidated.

We are indebted to Dr P. Monaghan for electron microscopy and to Mrs Diana Mitchell and her staff for histology.

### REFERENCES

ARAFAH, B. M., GULLINO, P. M., MANNI, A. & PEARSON, O. H. (1980) Effect of ovariectomy on hormone receptors and growth of *N*-nitrosomethylurea-induced mammary tumors in the rat. *Cancer Res.*, **40**, 4628.

BAUM, M. (1977) The curability of breast cancer. In *Breast Cancer Management—Early and Late.* (Ed. Stoll). London: William Heinemann.

BRUNSON, K. W., BEATTIE, G. & NICOLSON, G. L. (1978) Selection and altered properties of brain-colonising metastatic melanoma. *Nature*, **272**, 543.

BRUNSON, K. W. & NICOLSON, G. L. (1978) Selection and biologic properties of malignant variants of a murine lymphosarcoma. *J. Natl Cancer Inst.*, **61**, 1499.

DIXON, B. & SPEAKMAN, H. (1979) Local recurrence and metastasis of excised breast carcinoma in the rat. *J. R. Soc. Med.*, **72**, 572.

EASTY, D. M., EASTY, G. C., CARTER, R. L., MONAGHAN, P. & BUTLER, L. J. (1981) Ten human carcinoma cell lines derived from squamous carcinomas of the head and neck. *Br. J. Cancer*, **43**, 772.

FIDLER, I. J. (1973) Selection of successive tumour lines for metastasis. *Nature* (*New Biol.*), **242**, 148.

FIDLER, I. J. & KRIPKE, M. L. (1977) Metastasis results from pre-existing variant cells within a malignant tumor. *Science*, **197**, 893.

GIAVAZZI, R., ALESSANDRI, G., SPREAFICO, F., GARATTINI, S. & MANTOVANI, A. (1980) Metastasizing capacity of tumour cells from spontaneous metastases of transplanted murine tumours. *Br. J. Cancer*, **42**, 462.

GULLINO, P. M., PETTIGREW, H. M. & GRANTHAM, F. H. (1975) *N*-nitrosomethylurea as a mammary gland carcinogen in rats. *J. Natl Cancer Inst.*, **54**, 40.

KERBEL, R. S., TWIDDY, R. R. & ROBERTSON, D. M. (1978) Induction of a tumor with greatly increased metastatic growth potential by injection of cells from a low-metastatic H-2 heterozygous tumor cell line into an H-2 incompatible parental strain. *Int. J. Cancer*, **22**, 583.

KIM, U. (1979) Factors influencing metastasis of breast cancer. In *Breast Cancer: Advances in*

*Research and Treatment*. Vol. 3, (Ed. McGuire). New York: Plenum Pub. Corp. p. 1.

KREIDER, J. W., BARTLETT, G. L. & PURNELL, D. M. (1976) Suitability of rat mammary adenocarcinoma 13762 as a model for BCG immunotherapy. *J. Natl Cancer Inst.*, **56**, 797.

KRIPKE, M. L., GRUYS, E. & FIDLER, I. J. (1978) Metastatic heterogeneity of cells from an ultraviolet light-induced murine fibrosarcoma of recent origin. *Cancer Res.*, **38**, 2962.

LEWKO, W. M., LIOTTA, L. A., WICHA, M. S., VONDERHAAR, B. K. & KIDWELL, W. R. (1981) Sensitivity of *N*-nitrosomethylurea-induced rat mammary tumors to cis-hydroxyproline, an inhibitor of collagen production. *Cancer Res.*, **41**, 2855.

LINDSEY, W. F., DAS GUPTA, T. K. & BEATTIE, C. W. (1981) Influence of the estrous cycle during carcinogen exposure on nitrosomethylurea-induced rat mammary carcinoma. *Cancer Res.*, **41**, 3857.

MCCORMICK, D. L., ADAMOWSKI, C. B., FIKS, A. & MOON, R. C. (1981) Lifetime dose-response relationships for mammary tumor induction by a single administration of *N*-methyl-*N*-nitrosourea. *Cancer Res.*, **41**, 1690.

MCGUIRE, W. L., CARBONE, P. P., SEARS, M. E. & ESCHER, G. C. (1975) Estrogen receptors in human breast cancer: An overview. In *Estrogen Receptors in Human Breast Cancer* (Eds. McGuire *et al.*). New York: Raven Press. p. 1.

MOLLENHAUER, H. H. (1964) Plastic embedding mixtures for use in electron microscopy. *Stain Technol.*, **39**, 111.

NERI, A., RUOSLAHTI, E. & NICOLSON, G. L. (1979) Relationship of fibronectin to the metastatic behaviour of rat mammary adenocarcinoma cell lines and clones. *J. Supramolec. Struct.*, Suppl. **3**, 181.

NICOLSON, G. L., BRUNSON, K. W. & FIDLER, I. J. (1978) Specificity of arrest, survival and growth of selected metastatic variant cell lines. *Cancer Res.*, **38**, 4105.

RAZ, A. & HART, I. R. (1980) Murine melanoma: a model for intracranial metastasis. *Br. J. Cancer*, **42**, 331.

ROSE, D. P., PRUITT, B., STAUBER, P., ERTURK, E. & BRYAN, G. T. (1980) Influence of dosage schedule on the biological characteristics of *N*-nitrosomethylurea-induced rat mammary tumors. *Cancer Res.*, **40**, 235.

SIEGAL, G. P., BARSKY, S. H., TERRANOVA, V. P. & LIOTTA, L. A. (1981) Stages of neoplastic transformation of human breast tissue as monitored by dissolution of basement membrane components. *Invasion Metastasis*, **1**, 54.

SUZUKI, N., WITHERS, H. R. & KOEHLER, M. W. (1978) Heterogeneity and variability of artificial lung colony-forming ability among clones from mouse fibrosarcoma. *Cancer Res.*, **38**, 3349.

TAO, T.-W., MATTER, A., VOGEL, K. & BURGER, M. M. (1979) Liver-colonizing melanoma cells selected from B-16 melanoma. *Int. J. Cancer*, **23**, 854.

TURCOT-LEMAY, & KELLY, P. A. (1980) Characterization of estradiol, progesterone and prolactin receptors in nitrosomethylurea-induced mammary tumors and effect of anti-estrogen treatment on the development and growth of these tumors. *Cancer Res.*, **40**, 3232.

WILLIAMS, J. C., GUSTERSON, B., HUMPHREYS, J. & 4 others (1981) *N*-methyl-*N*-nitrosourea-induced rat mammary tumors. Hormone responsiveness but lack of spontaneous metastasis. *J. Natl Cancer Inst.*, **66**, 147.

WILLMOTT, N., AUSTIN, E. B. & BALDWIN, R. W. (1979) Comparative studies of the metastatic potential of three transplantable rat mammary carcinomas of spontaneous origin. *Br. J. Exp. Pathol.*, **60**, 499.